# Innate Immunity against *Cryptococcus*, from Recognition to Elimination

**DOI:** 10.3390/jof4010033

**Published:** 2018-03-07

**Authors:** Althea Campuzano, Floyd L. Wormley

**Affiliations:** 1 Department of Biology, The University of Texas at San Antonio, San Antonio, TX 78249, USA; althea.campuzano@my.utsa.edu; 2 South Texas Center for Emerging Infectious Diseases, The University of Texas at San Antonio, San Antonio, TX 78249, USA

**Keywords:** *Cryptococcus neoformans*, *Cryptococcus gattii*, *Cryptococcus deneoformans*, host–pathogen interactions, pattern recognition receptors (PRRs), innate immune response, pathogen-associated molecular patterns (PAMPs), C-type lectin receptors (CLRs), Toll-like receptors (TLRs), NOD-like receptors (NLRs)

## Abstract

*Cryptococcus* species, the etiological agents of cryptococcosis, are encapsulated fungal yeasts that predominantly cause disease in immunocompromised individuals, and are responsible for 15% of AIDS-related deaths worldwide. Exposure follows the inhalation of the yeast into the lung alveoli, making it incumbent upon the pattern recognition receptors (PRRs) of pulmonary phagocytes to recognize highly conserved pathogen-associated molecular patterns (PAMPS) of fungi. The main challenges impeding the ability of pulmonary phagocytes to effectively recognize *Cryptococcus* include the presence of the yeast’s large polysaccharide capsule, as well as other cryptococcal virulence factors that mask fungal PAMPs and help *Cryptococcus* evade detection and subsequent activation of the immune system. This review will highlight key phagocyte cell populations and the arsenal of PRRs present on these cells, such as the Toll-like receptors (TLRs), C-type lectin receptors, NOD-like receptors (NLRs), and soluble receptors. Additionally, we will highlight critical cryptococcal PAMPs involved in the recognition of *Cryptococcus*. The question remains as to which PRR–ligand interaction is necessary for the recognition, phagocytosis, and subsequent killing of *Cryptococcus*.

## 1. Introduction

### 1.1. Cryptococcosis

*Cryptococcus* was first identified as a human pathogen as early as 1894, by Otto Busse. Disease due to this fungal pathogen was rare, but *Cryptococcus* became known as a significant opportunistic fungal pathogen, causing life-threatening infections of the central nervous system (CNS) during the AIDS epidemic [1]. The medically relevant species, *Cryptococcus neoformans (C.n.)*, *Cryptococcus gattii* (*C.g.*), and *Cryptococcus deneoformans* (*C.d.*), vary in their pathobiology, but are treated similarly by clinicians [2,3]. *C. neoformans*, *C. gattii*, and *C. deneoformans* were previously known as *C. neoformans* var. *grubii*, *C. gattii*, and *C. neoformans* var. *neoformans*, respectively [4]. Despite antifungal treatment, the acute mortality rate caused by the dissemination of *Cryptococcus* to the CNS results in 223,100 cases of cryptococcal meningitis and 181,100 deaths worldwide [5].

*Cryptococcus* is associated with causing disease in hosts with impaired immunity. Cryptococcosis is an AIDS-defining illness, and remains the most fatal fungal disease among AIDS patients worldwide [6]. This organism also impacts immunocompetent individuals. Pirofski and Casadevall recently reviewed the outcome of host–*Cryptococcus* interactions in the damage response framework [7]. The emergence of *Cryptococcu*s-related immune reconstitution inflammatory syndrome (IRIS) following antiretroviral therapy (ART) demonstrated the impact of host-induced inflammation, leading to the aggravation of disease [8]. Additionally, Neal et al. used a murine model of cryptococcal meningoencephalitis to demonstrate that disease exacerbation is due to the CD4^+^ T cell-mediated immune response, instead of the fungal burden in the CNS [9]. Therefore, defining the proper immune surveillance for *Cryptococcus* by the host’s immune response is necessary to reduce morbidity and mortality due to systemic fungal infections.

*Cryptococcus* is found ubiquitously in the environment as an encapsulated budding yeast, and exposure can be detected during the early weeks of life [10]. Inhalation is the primary route of infection; however, *Cryptococcus* has a propensity to disseminate from the lungs to the CNS, particularly when cell-mediated immunity is compromised. Protective immunity against *Cryptococcus* is dependent on recognition, control, and proper interaction by and with cells of the innate and cell-mediated immune response. Historically, pro-inflammatory Th1-type immune responses are associated with protection against cryptococcosis, whereas Th2-type immune responses are associated with exacerbation of disease [11,12,13,14]. *C. neoformans* and *C. gattii* appear to skew the immune response towards a nonprotective Th2-type response, leading to its escape from the phagosome, proliferation within phagocytes, and aggravation of disease [15,16]. Mukaremera and Nielsen recently published a comprehensive review on the role of the adaptive immune response [17]. This review will highlight the role of the innate cellular immune members, the arsenal of PRRs utilized by these cells to detect *Cryptococcus*, as well as how the yeast is able to evade detection by the host.

### 1.2. Host Immune Response

Cellular components of the innate immune response—macrophages, dendritic cells (DCs), neutrophils, and monocytes—are on the front line to defend against fungal pathogens. Control or eradication of fungal pathogens begins with the recognition and phagocytosis of the yeast by these phagocytic cells of the innate immune response. Once the yeast is internalized within a phagosome, phagosome–lysosome fusion occurs, resulting in inflammasome activation, acidification of the phagosome-lysosome, and subsequent degradation of the yeast. Additionally, phagocytic cells produce reactive oxygen and nitrogen species (ROS and RNS, respectively), cytokines, and chemokines, as well as presenting antigens to T cells that then directs the adaptive immune response [18]. Activation of the protective Th1-type immune response is associated with the production of interferon-γ (IFN-γ), interleukin-2 (IL-2), and IL-12 [11,12,14,19]. Mice with gene disruption of Th1-type cytokines, such as IFN-γ, IL-12, IL-18, and tumor necrosis factor alpha (TNF-α), are more susceptible to cryptococcal infections as compared with WT mice [20,21,22]. The Th17-type response is associated with the production of IL-6, IL-17A, IL-21, IL-22, and transforming growth factor (TGF)-β, and is associated with anti-cryptococcal immune responses [23,24,25,26,27]. However, while IL-17A contributes to protection, it is not required for the protection and eventual eradication of *Cryptococcus* in mice [28]. On the other hand, Th2-type cytokine responses are associated with IL-4, IL-5, and IL-13 cytokine production, and are involved in the recruitment of eosinophils and the exacerbation of disease [13,29]. Virulence factors, such as urease and laccase, that are present in pathogenic *Cryptococcus* strains are able to modulate its environment towards a nonprotective Th2-type response in murine studies [14,16,30,31]. Studies evaluating the host immune responses in mice given an experimental pulmonary infection with a genetically modified *C. neoformans* clinical isolate, H99, that secretes murine IFN-γ, denoted H99γ, showed that Th1- and Th17-type responses are required for protection [19,32]. Therefore, the cytokine response plays a significant role in protective antifungal immunity, and early detection and clearance by the innate immune response is necessary to prevent the dissemination of *Cryptococcus*.

## 2. Innate Immune Cells

### 2.1. Macrophages

Following the inhalation of yeast or desiccated basidiospores, the incoming pathogens are detected by lung-resident macrophages, which represent more than 90% of leukocytes in the bronchoalveolar lavage of healthy hosts [33]. Alveolar and infiltrating macrophages initiate anti-cryptococcal immune responses by recognizing and engulfing the yeasts. Macrophages play a critical role in regulating the disease outcome by aiding in fungal clearance or dissemination, depending on their activation status. These phagocytes are highly versatile and are associated with two critical phenotypes: M1, or classically activated macrophages; and M2, or alternatively activated macrophages [34]. M1 macrophages are critical for the eradication of *Cryptococcus* through the production of ROS and RNS. In contrast, M2 macrophages support intracellular survival and cryptococcal proliferation, resulting in persistence of the infection [35]. These phenotypes are defined by macrophage cytokine responses and their expression of specific extracellular receptors. M1 markers include: inducible nitric oxide (iNOS or NOS2); suppressor of cytokine signaling 3 (SOCS3); and the chemokines C-X-C motif 9 (CXCL9), CXCL10, and CXCL11 [35,36]. M2 markers include Arginase-1 (Arg-1), found in inflammatory zone 1 (Fizz1), chitinase and the chitinase-like molecule (Chi3l3, also known as Ym1), and the extracellular receptor CD206 (or Mannose receptor, MR) [37]. iNOS and Arginase-1 compete for the same substrate: l-arginine. M1 macrophages will metabolize arginine via NOS2 to produce nitric oxide and citrulline, while M2 macrophages produce urea and ornithine [38].

Polarization towards M1/M2 macrophages is dependent on the cytokine microenvironment during infection [39]. The cytokine profile required to modulate M1 macrophage activation during a pulmonary infection with *Cryptococcus* is dependent on an IFN-γ-dominant cytokine milieu, while, in contrast, an IL-4 and/or IL-13-dominant cytokine milieu leads to the development of M2 macrophages and intracellular proliferation of yeast [40,41,42]. IFN-γ production by Th1-type CD4^+^ T cells and NK cells stimulates M1 macrophage activation via signal transducer and activator of transcription 1 (STAT1) [43]. Protective antifungal immunity is associated with STAT1 signaling, which is required for M1 macrophage activation and induction of protection against fungal pathogens [44,45]. However, IL-17A cytokine production is not required for M1 macrophage activation [46]. *Cryptococcus* is capable of affecting the polarization of macrophages towards a nonprotective M2 phenotype via the heat shock protein 70 homolog Ssa1, by inducing IL-4 and IL-13 production and the expression of CD206 (MR) and Arginase-1 in bone marrow-derived macrophages [47].

### 2.2. Dendritic Cells

Dendritic cells (DCs) are sentinel cells of the innate immune system, professional antigen-presenting cells (APCs), and bridges between the innate and adaptive immune responses. DCs populate the airways, sensing for invading microorganisms. Phagocytosis and subsequent killing of *Cryptococcus* by DCs is enhanced by complement or antibody opsonization [48,49]. Phagosome maturation occurs by phagosome fusion and fission with endosomes, resulting in phagolysosome maturation. Following phagocytosis, cryptococci are compartmentalized within the phagolysosome, and degraded by oxidative and nonoxidative mechanisms [50,51]. Cathepsin B present within the phagolysosome forms pores in the cell wall, resulting in the lysis of *Cryptococcus* [52].

DCs undergo the enhancement in expression of several costimulatory molecules (CD40, CD80, and CD86), maturation, and then present processed antigen to naïve T cells via major histocompatibility complex II (MHCII) [53,54]. DC maturation in the presence of INF-γ results in the formation of IL-12-producing DCs, which can subsequently produce cytokines, such as IFN-γ, that drive Th1-type responses [55]. CD86 expression with OX40L can induce Th2 cells that secrete the anti-inflammatory cytokines IL-4, IL-5, and IL-13, which are associated with the recruitment of eosinophils [50]. T cell activation provides the necessary signals required for the production of effector cytokines leading to a Th1-type response. In the absence of macrophages and DCs, PMNs and B cells accumulate in the lung but are unable to control the fungal infection, and their increasing presence is associated with excess damage to the host [56]. *Cryptococcus* evades detection by DCs through the production of the virulence factor urease, which promotes the accumulation of immature dendritic cells, rendering these APCs ineffective [16]. Furthermore, the depletion of TNF-α rendered mice more susceptible to *C. neoformans* pulmonary infections, leading to the alternative activation of DCs that cannot effectively clear fungi [57]. Additionally, *C. gattii* is able to suppress host responses in DCs, resulting in suppressed TNF-α levels [58].

### 2.3. Neutrophils

Neutrophils, or polymorphonuclear leukocytes (PMNs), are antigen-presenting cells present in the lungs, which have antifungal capabilities. In vitro, granulocyte colony stimulating factor (G-CSF) or granulocyte-macrophage colony stimulating factor (GM-CSF) enhances neutrophil anti-cryptococcal activity [59]. Recruitment of PMNs to *Cryptococcus* requires C5a-C5aR activation, which initiates mitogen-activated protein kinase (MAPK) members—extracellular signal-regulated kinases (ERK) and p38—resulting in pro-inflammatory cytokine production [60]. p38 inhibition significantly decreased the infiltration of PMNs and also inhibited cryptococcal killing. Complement C3 and CD11b expression led to the production of leukotriene B4, a migratory and activating eicosanoid, resulting in the swarming of *Cryptococcus* by neutrophils [61]. The capsular component glucuronoxylomannan (GXM) displays chemotactic activity in neutrophils; however, GXM is, conversely, also able to inhibit neutrophil migration and phagocytosis [62,63,64,65]. Specifically, O-acetylation of GXM is responsible for inhibiting PMN migration during infection [66]. GXM alone was also unable to induce neutrophil extracellular traps (NETs). In contrast, glucuronoxylomannogalactan (GXMGal) and an acapsular strain of *C. neoformans* (CAP67) induced the NET formation required for clearance, due to its fungicidal activity [67]. Human PMNs have anti-cryptococcal activity via nonoxidative and oxidative mechanisms, whereas respiratory burst somewhat reduced the antifungal activity of PMNs [68]. Neutrophil depletion in an experimental intratracheal infection model of cryptococcosis, as well as in mice infected with the *C. neoformans* strain H99γ, showed increased survival, demonstrating that neutrophil ablation did not affect the fungal burden, thereby indicating that they are not required for pulmonary cryptococcal clearance [69,70]. Furthermore, several studies have noted that the accumulation of neutrophils accompanies increased fungal burden during pulmonary infections [45,71,72,73]. Therefore, the role of neutrophils during the protective immune responses against *Cryptococcus* is complex.

## 3. Arsenal of Pattern Recognition Receptors

The phagocytic cells previously discussed are capable of detecting highly conserved PAMPs via germ-line encoded PRRs [18]. To date, three different classes of PRRs involved in pathogen clearance have been well characterized. These PRR members which are responsible for sensing for the presence of invading microorganisms include transmembrane Toll-like receptors (TLRs) and C-type lectin receptors (CLRs), as well as cytosolic receptors, such as NOD-like receptors (NLRs).

### 3.1. Toll-Like Receptors

Toll receptors that are homologous to mammalian TLRs were first identified in *Drosophila* as playing a major part in their development and protection against *Aspergillus* and other fungal species [74]. To date, TLRs are the most extensively studied family of PRRs, with 13 members characterized. These receptors are now associated with the recognition of bacteria and viruses as well as fungal pathogens and play a significant part in immunity as they are capable of modulating both pro-inflammatory and ant-inflammatory responses. TLRs are composed of an extracellular domain containing leucine-rich repeat motifs, with a cytoplasmic tail comprising a Toll/interleukin-1 receptor (TIR) domain. Recognition of PAMPS by TLRs initiates signal transduction cascades associated with the adaptor molecule myeloid differentiation primary response protein 88 (MyD88), with the exception of TLR3, and in part for TLR4 (MyD88 independent activation) [75,76]. Signal activation induces mitogen-activated protein kinases (MAPKs) and nuclear factor kappa beta (NFκB), activating various inflammatory genes, including interferon regulatory factors 5 and 7, (IRF5 and IRF7), AP-1, and NFκB, resulting in pathogen clearance [18,77].

TLR2 and TLR4 have gained much attention owing to their ability to recognize various pathogen ligands, particularly cell wall-associated ligands. These extracellular receptors are expressed on innate immune cells, including neutrophils, monocytes, macrophages, and DCs [78]. β-glucans that are expressed in the cell wall of several fungal pathogens are recognized by TLR2; however, *Cryptococcus* is able to mask the β-glucan layer with its capsule [79,80,81]. The role of TLR2 during the protective immune response to cryptococcosis varies, as the response is dependent on strain and capsule variability. A robust role for TLR2 recognition was shown in mice infected with *C. neoformans* strain H99, *C. deneoformans* strain B3501, and the acapsular strains 145A and CAP67 [82,83]. Contrary to those results, Nakamura showed that there was no significant difference in immune responses in TLR2-deficient mice as compared to WT mice during infection with *C. neoformans* strains H99 and YC-13, as demonstrated in Table 1 [83,84].

GXM is recognized by TLR2 and TLR4 as well as CD14 and CD18 in vitro, but these receptors are not required for serum clearance in vivo [85]. TLRs are also able to form heterodimers, such as TRL1/2 and TLR2/6, that can recognize the major capsular component GXM of *Cryptococcus* [86]. TRL4 becomes activated by O-linked mannans in *C. neoformans* in vitro. Nonetheless, despite this initial response, studies using TLR4-deficient mice demonstrated that TLR4 is dispensable in anti-cryptococcal immunity [82,84,87]. Furthermore, stimulation of microglial cells with the TLR agonists TLR1/2, TLR3, TLR4, and TLR9 increased pro-inflammatory cytokines following *C. neoformans* interaction; however, the significance of this is not clear [101].

**Figure 1:**
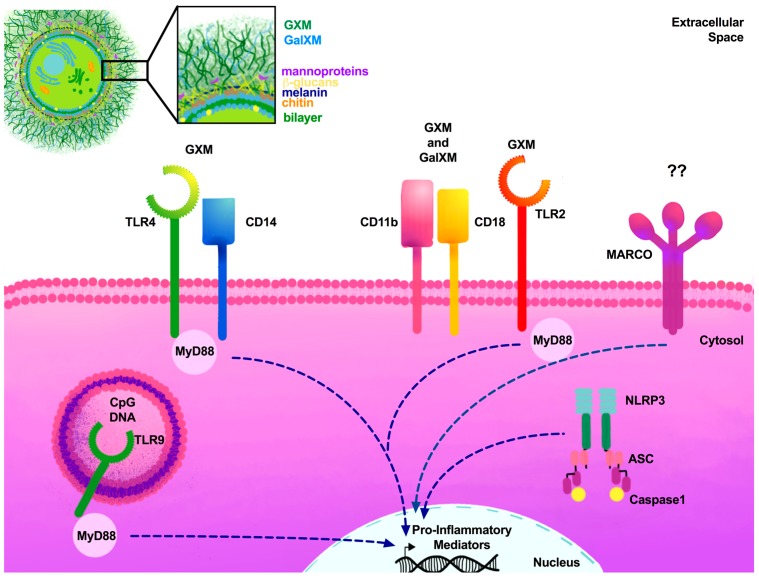
TLRs and scavenger receptors required for cryptococcal PAMPs. *Cryptococcus* species contain a large polysaccharide capsule made up of GXM and GalXM. Extracellular receptors present in myeloid cells recognize GXM and GalXM via TLRs. TLR4 forms heterodimers with other extracellular receptors, including CD14, in order to detect capsular polysaccharides. TLR2, CD11b, and CD18 are also able to detect the capsule. Intracellular phagosomal TLR9 recognizes unmethylated CpG motifs of *Cryptococcus.* The NLR member NLRP3 is crucial for processing internalized *cryptococci*. Following the recognition of cryptococcal PAMPs, the adaptor molecule MyD88 is essential for the induction of pro-inflammatory mediators. Dashed lines represent various MyD88-dependent and independent signaling pathways required for pro-inflammatory mediator activation. ?? = unknown cryptococcal ligand for MARCO receptor.

Once yeasts are phagocytized and internalized within the phagosome, fungal nucleic acids can be recognized, activating phagosomal TLRs. Numerous groups have demonstrated that TLR9 can recognize fungal genomic DNA and identify the unmethylated cytosine-phosphate-guanosine (CpG) motifs of *C. neoformans*, leading to phagosome recruitment and clearance of the fungal pathogen (Figure 1) [88,102,103,104]. DC cytokine responses were significantly altered in the absence of TLR9 [88]. Further experiments determined that poor disease outcome in the absence of TLR9 is attributed to failed DC activation via CCL7, affecting leukocyte recruitment [89]. While TLR9 continues to be investigated, crosstalk with other PRRs members may play a significant role in antifungal control. Recently, C-type lectin receptor Dectin-1-induced Syk activation was shown to result in the recruitment of TLR9 phagosomes containing β-1,3 glucan, *Aspergillus fumigatus*, and *C. albicans* [105]. Although various studies have been conducted to characterize the role of TLRs, the question still remains whether TLR2, TLR4, and heterodimers TLR1/2 and TLR2/6 are required for cryptococcal recognition and protective immune responses to cryptococcosis. Future studies may instead focus on the potential of TLR agonists or ligands as potential adjuvants for vaccine formulations to combat *C. neoformans* infections caused by the more virulent strains.

While specific TLR members may be dispensable during cryptococcal infections, the downstream adaptor molecule MyD88 is necessary for protection against *C. neoformans* infections [83]. MyD88^−/−^ mice are highly susceptible to fungal infections caused by *C. albicans*, *A. fumigatus*, *Coccidioides immitis*, and *Paracoccidioides brasiliensis*, as well as *C. neoformans*, compared to WT mice [106,107,108,109]. The significant role of MyD88 in fungal infections has been predominantly shown in mouse models. Recent studies in individuals who lacked functional MyD88 demonstrated that patients were more susceptible to bacterial infections, as compared to fungal infections [110,111]. These results may indicate that TLRs may not be the central players in antifungal immunity in humans, and that perhaps crosstalk to other PRR members may be required for optimal fungal recognition and eradication.

### 3.2. C-Type Lectin Receptors

C-type lectin receptors were first associated with the recognition of carbohydrate moieties present in pathogens through the transmembrane conserved motif known as the C-type lectin-like domain (CTLD). CLRs bind to carbohydrate moieties through one or more carbohydrate recognition domains (CRDs), and are expressed in myeloid cells, such as DCs and macrophages, as well as in lymphocytes [112]. CLRs have been categorized as either Dectin-1 or Dectin-2 clusters, based on the gene location in the chromosome [113]. Recognition by CLRs can result in the activation of immunoreceptor tyrosine-based activation motif (ITAM)-like/ITAM motifs present in the Fc-gamma receptor (FcγR), leading to the recruitment and activation of spleen tyrosine kinase (SYK) through protein kinase C delta (PKC-δ) and Vav proteins, thereby activating the caspase recruitment domain-containing protein 9 (CARD9)–B-cell lymphoma 10 (BCL10)–mucosa-associated lymphoid tissue lymphoma-translocation gene 1 (MALT1) scaffold complex [114,115,116,117]. Activation of the CARD9–BCL10–MALT1 signaling complex can serve as a scaffold for the activation of canonical NFκB and MAPK, which triggers macrophage activation, DC maturation, and ROS and cytokine production for antifungal responses [118]. Signaling through NFκB has been shown to be required for the pro-inflammatory and Th1-type cytokine responses necessary for the clearance of *C. neoformans* [119].

**Figure 2:**
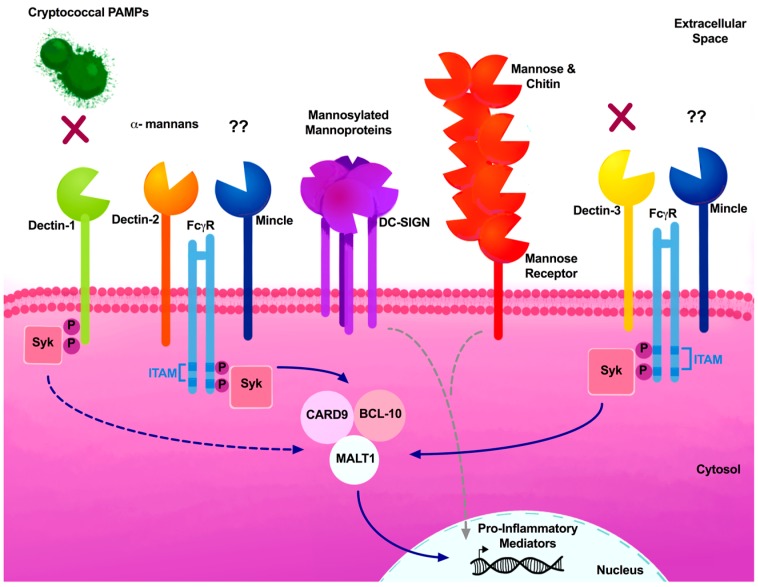
Critical CLR members associated with fungal PAMPs. CLR members Dectin-1 and Dectin-3 have been described as being dispensable during cryptococcal infections and are not required for recognition of *Cryptococcus* (X) in murine studies. Dectin-2 deficiency resulted in mice being skewed towards a debilitating Th2-type response. Multiple CRD-containing CLRs, such as DC-SIGN and CD209, recognize mannosylated mannoproteins, and the mannose receptor (MR) recognizes mannose and chitin. These receptors induce pro-inflammatory mediators in a ITAM independent manner (gray dashed lines). The Mincle receptor is poorly characterized during cryptococcosis and Mincle’s cryptococcal ligand continues to be elucidated (??). CLR signal transduction can utilize the ITAM sequence present in FcγRs. ITAM activation phosphorylation activates Syk, which can then directly (solid blue line) or indirectly (dashed blue lines) activate the adaptor molecule complex comprised of CARD9, MALT1 and BCL10. This complex can directly induce pro-inflammatory mediators (solid blue line).

CLRs are able to crosstalk with each other via the formation of heterodimeric complexes (Figure 2). Studies suggest that Dectin-3 interaction with FcγR requires Mincle for the formation of Dectin-3–Mincle heterodimers [120]. However, Miyake and colleagues demonstrated that Dectin-3 can interact with FcγR in the absence of the Mincle receptor [121], which contradicts initial immunoprecipitation studies of the interaction between FcRγ and Mincle receptors [122]. Lin and colleagues also demonstrated that Dectin-3 formed heterodimers with Dectin-2, and that these receptors had a higher affinity to α-mannan and hyphae of *C. albicans* [123]. These studies highlight the complexity of crosstalk between CLRs, as well as providing an explanation for the variability of results in receptor–ligand interactions.

The first CLR member to be characterized was Dectin-1 (dendritic cell-associated C-type lectin 1), as it was shown to have a significant role in the recognition of Zymosan and β-1,3-glucan fungal PAMPs [124,125,126]. Although the name implies that Dectin-1 is expressed in DCs, it can also be found on macrophages, neutrophils, and monocytes. Upon recognition of fungal β-glucans, Dectin-1 activates various signaling pathways independently of ITAM motifs, leading to the phagocytosis of fungi, activation of respiratory burst through ROS production, DC maturation, and induction of pro-inflammatory cytokines and chemokines [113]. β-glucans act as scaffolding structures present in a variety of fungi, including *Aspergillus*, *Candida*, *Histoplasma*, *Coccidioides*, *Penicillium*, *Pneumocystis*, and *Saccharomyces*, which can be recognized by Dectin-1 [79,81,127,128,129,130,131]. Although Dectin-1 plays a significant role in the detection of several fungi, Dectin-1 is dispensable for host defenses against *Cryptococcus* infections, owing to this pathogen’s ability to conceal the inner cell wall layer containing β-glucans beneath its polysaccharide capsule (Figure 2) [90]. In the absence of capsule formation, exposed β-glucans present in cryptococcal spores were recognized by Dectin-1 [132]. Recent studies conducted by Walsh et al. further characterized the CLR members required for spore recognition, and determined that although Dectin-1^−/−^ macrophages were not able to phagocytize spores as efficiently as Dectin-1^+/+^ macrophages, no differences in survival were observed between Dectin-1^−/−^ and Dectin-1^+/+^ mice challenged with *Cryptococcus* spores (Table 1) [91].

Dectin-2 (CLEC6A, CLEC4N) has a high affinity for high mannose and α-mannan structures, as expressed by *Candida* spp., *Aspergillus fumigatus*, *Trichophyton rubrum*, *Malassezia* spp., and *Saccharomyces* [93,133,134,135,136,137,138]. Dectin-2-deficient mice were shown to be more susceptible to *Candida* infections. In contrast, Dectin-2-deficient mice exposed to *Cryptococcus deneoformans* (formerly known as Serotype D, B3501), lacked effective protective Th1 or Th17 responses; instead, Th2-type cytokines such as IL-4 and IL-5 were more prevalent in their lungs, as compared with WT mice (Figure 2) [92]. Additionally, a screen using a NFAT-GFP reporter system to test for Dectin-2 recognition of pathogenic fungi showed that *Cryptococcus* does not recognize Dectin-2 (Table 1) [93]. The lack of recognition may be due to the expression of the polysaccharide capsule.

Dectin-3 (also known as MCL, CLEC4D and CLECSF8) was first identified through the PCR screening of macrophage-associated genes [139]. Dectin-3 transcripts were predominantly expressed in resident peritoneal macrophages, and at lower levels in the bone marrow. Dectin-3 activation leads to phagocytosis and the release of pro-inflammatory cytokines [121,122,140]. Dectin-3 knockout mice show no apparent phenotype when they are infected with *C. albicans* [122]; however, another investigative group found that Dectin-3-deficient mice were highly susceptible to *Candida* infections [123]. These contradicting results could be due to the variability of strains used, as well as variability in the inoculum. While Dectin-3 facilitates the recruitment of plasmacytoid DCs (pDCs) to the lungs during the protective immune response against pulmonary *C*. *neoformans* infection [94], Dectin-3 deficiency did not lead to increased susceptibility of mice to an experimental pulmonary infection of *C. neoformans* (Figure 2) [95]. Furthermore, pulmonary macrophages and DCs did not display any impairment in phagocytosis or killing in the absence of Dectin-3, demonstrating that Dectin-3 is dispensable against murine cryptococcal infections (Table 1) [95].

Mincle, or macrophage-inducible C-type lectin (CLEC4E, CLECSF9, C86253), was first recognized as a transcriptional target in activated macrophages, and not on resting macrophages [141]. Its expression is regulated by the constitutively expressed Dectin-3 receptor [121,142]. Yamasaki et al. determined that *Malassezia* is detected by Mincle and FcγR using a NFAT-GFP reporter assay, and that Mincle recognizes the glycerol glycolipids of *Malassezia* [96]. This study also evaluated the recognition of *Cryptococcus* spp. by Mincle, resulting in a lack of recognition by Mincle receptor in vitro (Table 1) [96].

Mannose receptor (MR, or CD206) is a non-ITAM-associated CLR that can recognize the terminal mannose residues of *C. albicans*, and *Pneumocystis*; and MR-deficient mice were also more susceptible to *C. neoformans* infections [98,143,144,145,146]. MR is involved in the binding and uptake of *Cryptococcus* by DCs, which is necessary for subsequent antigen presentation to CD4^+^ T cells. Blocking MR significantly decreased the uptake of *C. deneoformans* strain 613 [48,97]. Although MR does not possess the classical signaling motifs, MR is capable of inducing the production of IL-17, TNF-α, and MCP-1 [147]. Recently, Wagener et al. demonstrated that chitin, the second most abundant polysaccharide in nature and prevalent in fungi, is recognized by MR as well as by TLR9 and NOD2 [148].

Dendritic cell-specific ICAM-3-grabbing nonintegrins (DC-SIGN, CD209) are transmembrane receptors present in macrophages and dendritic cells [149,150]. DC-SIGN consists of a single calcium-dependent extracellular CTLD that recognizes heavily mannosylated cryptococcal mannoproteins (Figure 2 and Table 1) [98]. DC-SIGN is also associated with the internalization of antigens required for T cell presentation [151]. The role of DC-SIGN in fungal recognition was first associated with recognizing the N-terminal mannose residues of *C. albicans* that are transferred to the late endosomes and early lysosomes [152]. Recent studies evaluating polymorphisms in DC-SIGN demonstrated that there is a greater risk of pulmonary aspergillosis when DC-SIGN is compromised [153].

CARD9 is a critical adaptor protein that operates downstream of several CLRs, including Dectin-1, Dectin-2, Dectin-3, and Mincle. Because CARD9 is a central molecule to many overlapping signaling pathways, one can understand why its absence can result in high susceptibility to mucosal and systemic fungal infections. CARD9-deficient mice demonstrate the critical role for CARD9 in antifungal responses. CARD9^−/−^ mice are more susceptible to *C. albicans* infections [154], and humans carrying a CARD9 polymorphism also display enhanced susceptibility to *C. albicans* and *A. fumigatus* infections [155,156,157]. Challenge with other pathogenic fungi, such as *C. deneoformans* B3501, also showed that the deficient mice were highly susceptible to the fungal infections and were unable to clear the infections [158]. A recent review highlighted inborn errors in patients with CARD9 deficiency, demonstrating that CARD9 is solely associated with superficial and invasive fungal infections, rather than any susceptibility to bacterial or viral infections [159].

### 3.3. NOD-Like Receptors (NLRs)

Nucleotide-binding and oligomerization domain (NOD)-like receptors (NLRs) are cytoplasmic PRRs that play a crucial role in the innate immune response, and are capable of recognizing PAMPs and damage-associated molecular patterns (DAMPs). NLRs are subdivided into four subfamilies based on the N-terminal domain, such as the NLR pyrin domain (NLRP), and are associated with inflammasome assembly, signal transduction, transcription activation, and autophagy [160]. Recent studies have shown that NLRs have been implicated in sensing fungi. Once activated, NLRs are responsible for apoptosis-associated speck-like protein containing CARD (ASC)–caspase-1 inflammasome activation within macrophages and DCs producing IL-1β and IL-18 [161]. Mice that lack NLRP3 (Nalp3, Cryopyrin, CIAS1, and PYPAF1) were more susceptible to *Candida* infections, since the NLRP3 inflammasome aids in epithelial integrity, contributing to irritable bowel disease (IBD) in *C. albicans* yeast and hyphae morphologies, and leading to excess inflammation [162]. Internalization of the acapsular *C. neoformans* mutant CAP59 is able to activate the NLRP3 inflammasome, leading to the induction of IL-1β secretion, suggesting that the yeast capsule is able to mask itself following phagocytosis [99]. These observations were later tested by Chen and colleagues, who determined that the opsonization-mediated internalization of *C. neoformans* was also able to activate the canonical NLRP-3–ASC–caspase-1 inflammasome pathways via potassium efflux and membrane permeability (Table 1) [100].

### 3.4. Other Critical Receptors

Activation of the scavenger receptor macrophage receptor with collagenous structure (MARCO or SR-A6) on macrophages can mediate endocytosis as well as apoptosis [163]. Xu et al. recently characterized its role in antifungal immunity to *C. neoformans* (Figure 1). MARCO-deficient mice were associated with decreased leukocyte recruitment of Ly6^high^ monocytes and monocyte-derived DC (moDC); and decreased phagocytosis of alveolar macrophages of subsets CD103^+^, moDC^+^, and CD11b^+^ DC [164]. These results suggest that MARCO is required for initial recognition by the innate immune cells. Subsequent studies have evaluated the role of MARCO during the adaptive immune response to *Cryptococcus*, and showing that MARCO-deficient mice had better disease outcomes. MARCO-deficient mice were able to induce a Th1-driven immune response with increased production of INF-γ and TNF-α and increased M1 macrophage activation during *Cryptococcus* infection. These results are indicative of the role of MARCO in promoting the alternative activation of CD11b^+^ DCs that is detrimental to the host [165].

Macrophages express a variety of receptors, including complement receptor3 (CR3) and FcγR, which are required for the opsonization and phagocytosis of pathogens, including *Cryptococcus* [166,167]. Therefore, fungi will attempt to inhibit recognition by complement members in order to improve their survival. Antiphagocytic protein 1 (App1) in *C. neoformans* is able to bind to host CR2/CR3, thereby inhibiting phagocytosis by macrophages [168]. Additionally, recognition via classical complement activation is not required for protection against *C. gattii*. In the absence of C3 and Factor B, mice are significantly more susceptible to fungal infections, compared with WT mice [169]. Furthermore, previous studies have demonstrated that *C. gattii* alters its capsular GXM structure, thereby evading the innate immune response [170].

Galectin-3, an extracellular mammalian β-galactoside-binding protein, was recently characterized for its impact during *C. neoformans* infections [171]. Galectin-3 levels in serum are elevated in *C. neoformans*-infected mice, as well as in patients with cryptococcosis, both immunocompetent and HIV^+^, suggesting that *C. neoformans* drives Galectin-3 production by the host [171]. Additionally, Galectin-3 influenced cryptococcal growth and the stability of extracellular vesicles which are released by *Cryptococcus* into the extracellular environment, perhaps to suppress host immune responses [172].

## 4. Cryptococcal Cell Wall PAMPs

*Cryptococcus* possess several virulence factors that allow the yeast to evade host defense mechanisms, resulting in improved yeast survival and proliferation. Some critical virulence factors include the production of melanin and phospholipase B; but perhaps the most significant virulence factor is the polysaccharide capsule [173]. The capsule is able to mask ligands commonly detected by the immune response, and is composed of three major components: glucuronoxylomannan (GXM), galactoxylomannans (GalXM), and mannoproteins. GXM is the major component of the capsule, making up >90% of its mass (Figure 1). The composition of the capsule is heterogeneous, with the outer layer being more permeable, while the inner layer is rigid and compact [174]. The inner capsular layer thus prevents antibodies or complement detecting the cell wall.

The capsule is anchored by α-1,3-glucan linkages [175], while the cell wall membrane is comprised of β-1,3- and β-1,6-glucans as well as mannoproteins that act as a scaffold (Figure 1) [176]. The most inner membrane layer is comprised of chitin (GlcNAc polymer), and the deacetylated form, chitosan [177]. These are immunoreactive PAMPs that require masking by the polysaccharide capsule to evade detection. The cell wall components are synthesized intracellularly and exported to the extracellular space via vesicle-mediated secretion [178]. The secreted extracellular vesicles, known as ‘virulence bags’, also contain several virulence factors, including melanin, as well as cell wall components, such as GXM, that may deliver toxic payloads to phagocytic cells that ingest *Cryptococcus* yeasts [173,177]. Recent studies conducted by Stappers et al. identified the novel MelLec (CLEC1A) receptor, which recognizes melanin in *Aspergillus*; however, the role of MelLec in the recognition of *Cryptococcus* remains unknown [179].

Current studies have shown that the alteration of specific *Cryptococcus* genes associated with regulating cell wall development and capsule attachment results in increased host pro-inflammatory immune responses. The cryptococcal transcription factor Rim101 is a highly conserved pH-response regulator in several fungal pathogens, which also regulates the cell wall structure of *C. neoformans* [72]. Deletion of Rim101 alters the cell surface, resulting in exuberant pulmonary inflammatory responses and increased neutrophil recruitment to the lungs [72]. Rim101 regulates capsule attachment, leading to a thicker composition; the mutant displays variability in cell wall chitin and chitosan, leading to an exaggerated inflammatory response [180].

## 5. Concluding Remarks

As we continue to be exposed to fungi, we rely on our innate immune response to actively search out and identify incoming threats to our health. Macrophages play a significant role in the regulation of the disease outcome, as they can be skewed towards a protective M1 classically activated phenotype, or an M2 alternatively activated phenotype that is associated with increased pathogenesis during cryptococcosis. DCs aid in bridging the innate and adaptive arms of the immune system to modulate anti-cryptococcal immune responses. Neutrophils have played a significant role in protection against other fungal pathogens; however, animal models suggest that neutrophils contribute to disease progression and immune pathology [72], as previously mentioned in the damage response framework [7]. The innate immune cells possess PRRs that are critical for the detection and subsequent induction of protection against fungal pathogens via recognition of fungal PAMPs.

We have highlighted the major findings in *Cryptococcus* recognition by TLRs, CLRs, and NLRS. Due to redundancies in PAMP recognition by a myriad of PRRs, multiple signaling pathways may respond effectively to trigger appropriate antimicrobial and cytokine responses and phagocyte recruitment to aid in fungal clearance. Through the improvements of molecular techniques and in silico modeling, we have been able to identify several key polymorphisms associated with PRRs and their adaptor molecules MyD88 and CARD9 and understand their importance in protection against several fungal pathogens. In the future, further insights of mutations that predispose individuals to fungal infection will be further characterized, broadening our understanding even more.

We continue to improve our understanding of many of the interactions and signaling pathways involved with TLRs and CLRs. This review highlighted those PRRs that have been recently characterized as having a role in protection against *Cryptococcus*, as well as those strategies that *Cryptococcus* uses to remain elusive. Ultimately, our goal is to identify key PRRs and exploit PRR–PAMP interactions for the creation of future vaccines and/or therapies to combat cryptococcosis and other mycoses.

## Figures and Tables

**Table 1:** Pattern recognition receptor (PRR) and pathogen-associated molecular pattern (PAMP) identification and outcome.

PRRs	PAMP	Model System	Outcome	Citation
TLR2	GXM	TLR2 KO (C57B1/6)	TLR2 KO mice were more susceptible to experimental pulmonary, but not systemic, *Cryptococcus* infection.	[82]
			No significant difference in mortality observed between WT and TLR2 KO mice infected via i.p. inoculation. However, TLR2 KO mice experienced significant increases in fungal burden and decreases in pro-inflammatory cytokine responses.	[83]
			Limited role for TLR2 in host response to *C. neoformans*.	[84]
			TLR2 is not required for clearance of GXM found in serum.	[85]
TLR2/TLR1 and TLR2/TLR6	GXM	HEK293	GXM from various *Cryptococcus* serotypes were differentially recognized by TLR2/TLR1 and TLR2/TLR6 heterodimers expressed on TLR-transfected HEK293 cells.	[86]
TLR2/CD14	GXM	CHO cells	CHO cells transfected with both CD14 and TLR2 were not activated in response to *Cryptococcus* GXM.	[87]
TLR4/CD14	GXM	CHO cells	CHO cells transfected with both CD14 and TLR4 were activated in response to *Cryptococcus* GXM.	[87]
TLR4	GXM	C3H/HeJ	No significant difference in mortality observed in C3H/HeN mice compared to C3H/HeJ mice with loss of functional TLR4 receptor.	[83]
		TLR4 KO (C57B1/6)	No significant difference in pulmonary pro-inflammatory cytokine production in infected TLR4 KO mice compared to WT mice.	[84]
			TLR4 is not required for clearance of GXM found in serum.	[85]
TLR9	Cryptococcosis DNA	TLR9 KO (C57B1/6)	TLR9 KO mice were more susceptible to experimental pulmonary cryptococcosis.	[88]
			TLR9 KO mice showed increased fungal burden and decreased Rh1-type cytokine responses.	[89]
Dectin-1 (CLEC7A, CLECSF12, CD369)	β-glucans	Dectin-1 KO (C57B1/6)	Dectin-1 receptor is dispensable for recognition of cryptococcal yeast and spores.	[90,91]
Dectin-2 (CLEC6A, CLEC4N)	α-mannans	Dectin-2 KO (C57B1/6)	Dectin-2 KO mice lacked effective protective Th1 or Th17 responses and, interested, demonstrated elevated Th2-type cytokine responses.	[92]
		NFAT-GFP reporter cells	Dectin-2 NFAT-GFP reporter system did not recognize *Cryptococcus*.	[93]
Dectin-3 (MCL, CLEC4D, CLECSF8)	α-mannans?	Dectin-3 KO (C57B1/6)	Dectin-3 facilitates recruitment of pDCs to the lungs. However, Dectin-3 is dispensable for recognition and phagocytosis of *Cryptococcus* by pulmonary macrophages and DCs.	[94,95]
Mincle (CLEC4E, CLECSF9)	Glycerol-glycolipid	NFAT-GFP reporter cells	Mincle NFAT-GFP reporter system did not recognize *Cryptococcus*.	[96]
Mannose Receptor (CD206)	Mannose and chitin	Mannose Receptor KO (C57B1/6)	Mannose receptor expression on DCs were necessary for phagocytosis of *Cryptococcus* and stimulation of CD4^+^ T cells.	[97]
DV-SIGN (SIGNR, CD209)	mannoprotein	K562 cell line	Transfected DC-SIGN cells had an increased affinity to cryptococcal mannoproteins.	[98]
NLRP3	Internalized pathogens	NLRP3 KO (C57B1/6)	NLRP3 is activated in the presence of acapsular and capsular *Cryptococcus*, resulting in internalization and effective cryptococcal killing.	[99,100]

CHO = Chinese hamster ovary cell lines; DCs = dendritic cells; pDCs = plasmacytoid DCs; i.p. = intraperitoneal; GXM = glucuronoxylomannan; HEK = human embryonic kidney cell lines; KO = knockout; NFAT = nuclear factor of activated T cells; WT = wild-type; α-mannans? = recognition of *C. albicans* α-mannans by Dectin-3 continues to be characterized, while the Cryptococcal ligand is still unknown.
